# Molecules, Water, and Radiant Energy: New Clues for the Origin of Life

**DOI:** 10.3390/ijms10041419

**Published:** 2009-03-27

**Authors:** Gerald H. Pollack, Xavier Figueroa, Qing Zhao

**Affiliations:** Department of Bioengineering, Box 355061, University of Washington, Seattle WA, 98195, USA; E-Mails: xavierfm@u.washington.edu (X.F.); zaibingyifang@hotmail.com (Q.Z.)

**Keywords:** Colloids, radiant energy, exclusion zones, gels, origin, life, water, unstirredlayer.11

## Abstract

We here examine the putative first step in the origin of life: the coalescence of dispersed molecules into a more condensed, organized state. Fresh evidence implies that the driving energy for this coalescence may come in a manner more direct than previously thought. The sun’s radiant energy separates charge in water, and this free charge demonstrably induces condensation. This condensation mechanism puts water as a central protagonist in life rather than as an incidental participant, and thereby helps explain why life requires water.

## 1. Introduction

Many years ago in his classic book, *Origin of Life*, Oparin concluded that life began as a coascervate [1]. The term “coascervate” is little used today. It refers to a condensed colloidal state that bears much resemblance to the gel state, which has likewise been argued to harbor life’s origin [2]. Both states reflect a condensation of matter — a necessary condition for life’s origin.

This paper explores the mechanism of accumulation of molecules into the condensed state. The focus is on the forces responsible for driving condensation, and the energy source underlying those forces. Recent work has provided fresh clues which derive primarily from the recently revealed nature of interfacial water, i.e., water adjacent to hydrophilic surfaces. Such water turns out to have features remarkably different from bulk water, and these features appear to underlie the attraction and repulsion of substances. Oppositely charged substances are attracted naturally; but the above-mentioned features go on to explain why even like-charged substances are attracted to one another to form condensed coascervates — thereby permitting multiple repeats of the same molecule to condense into a proto-cell.

## 2. Discussion

### 2.1. Interfacial Water and Exclusion Zones

Exploring the nature of water at interfaces, we found several years ago that colloidal and molecular solutes were profoundly excluded from regions adjacent to certain hydrophilic surfaces [3]. These solute-free regions extended to distances as much as several *hundred* micrometers from the surface. Numerous controls showed that the observed exclusion was not the trivial result of some unsuspected artifact [3], and by now the simple experiments showing such long-range exclusion have been repeated in many laboratories and were in fact first reported four decades ago [4]. We term these apparently solute-free regions “exclusion zones.”

Exclusion zones are observed next to many surfaces including hydrogels, hydrophilic polymers, monolayers, ion-exchange beads and biological tissues. They exclude a diverse array of solutes of various size, type, and polarity. Hence, the exclusion phenomenon is a general feature of water adjacent to hydrophilic surfaces [5,6].

To test whether the physical properties of exclusion-zone water differ from those of bulk water, six methods have so far been applied. They include the following: NMR, infrared, and birefringence imaging; and, measurements of electrical potential, viscosity, and UV-VIS absorption spectra. The results confirm that the interfacial zone differs physically from the bulk zone, and that the former is a distinct, less mobile, more ordered phase of water that can coexist with the contiguous bulk phase [5,7].

### 2.2. Charge Separation and Energy

Of the aforementioned exclusion-zone observations, a particularly significant one is charge separation. Although the overall net charge may remain zero, water in the exclusion zone is negatively charged, while the bulk-water region beyond the exclusion zone contains positive charges. The potential difference between the two regions is 100 to 200 mV, depending on the particular hydrophilic surface. Electrodes inserted into the respective zones and connected through a resistor show ample, persisting, current flow [7].

Charge separation within water itself may seem counterintuitive, but it is surprisingly common. In cloud water for example, evidence of charge separation lies in the consequent lightning discharges, 80% of which occur from cloud to cloud. In the laboratory, the famous Kelvin water-dropper experiment shows electric discharge between juxtaposed bodies of pure water, again indicating waterbased charge separation. (Modern incarnations abound– e.g., see final six minutes of <http://www.youtube.com/watch?v=RQX8I9ZWtPQ&feature=relatedLecture>).

Charge separation in water has also been demonstrated in our laboratory. In a chamber filled with pure water, electrodes placed at either end of the chamber and driven at low voltage to pass current through the water, create ample charge buildup: the half of the water bath nearest the anode remains positive, while the half nearest the cathode remains negative, even well after the electrical driver has been disconnected [8]. Such stored water-based charge turns out to be largely recoverable [9].

These three examples offer precedent for charge separation in water, and give credence to the similar phenomenon observed in water near hydrophilic surfaces.

For building these charged exclusion zones, the required energy apparently comes from light. We found that incident radiant energy, including UV, visible, and near-infrared wavelengths, induces exclusion-zone growth. IR is especially effective: at wavelength 3.1 μm, a ten-minute exposure to incident radiation — weak enough to elevate water temperature by no more than 1°C — causes the exclusion-zone width to increase by up to four times [10]. This is an impressive increase for a relatively modest energy input, and it goes without saying (see below) that such solar-based or geothermal-based energy is likely to have relevance for the origin of life.

The molecular mechanism by which the exclusion zone expands is not fully understood. Incident photons must interact in some way with bulk water to build this zone. Water is naturally dissociated to some extent into H^+^ and OH^−^ (the basis of pH measurement); and, it is possible that incident photons enhance this natural dissociation near hydrophilic surfaces. The negatively charged moiety would then go on to build the negatively charged exclusion zone, leaving the bulk zone positively charged with protons or hydronium ions. In effect, this process amounts to a light-energy driven proton pump — a process surely useful for an incipient cell. In fact, the process is being exploited for obtaining clean electrical energy (patent pending).

Photons from sunlight or geothermal sources, then, may exert an effect that goes well beyond mere heating. Solar energy incident on water evidently separates charge. Such light-induced near-surface water splitting is reminiscent of what happens in reaction centers as the first step of photosynthesis. Hence, the charge-separation phenomenon identified here may be something akin to an initial step in photosynthesis — a kind of “generic” first step that occurs next to many hydrophilic surfaces rather than only next to those specific to green plants or bacteria. This process could represent not only a potentially significant energetic pathway that may be broadly relevant for nature, but also as we argue below, a central protagonist for the origin of life.

### 2.3. Strength of Evidence on Interfacial Water

To those unfamiliar with surfaces and water, the claims above may seem extraordinary. Given the intensity of scientific activity, how could it be possible that others might have failed to come upon a finding seemingly so simple?

In fact, others have. Many investigators have observed similar phenomena. A half-century-old review cited more than 100 references showing that for many liquids including water, near-surface zones differ from regions farther away in their physical chemical properties [11]. These zones project hundreds of microns from the surface. And, in his seminal book on life’s origin, Oparin repeatedly cites the work of Bungenberg de Jong [12], demonstrating extensive water structuring around colloidal particles of gum arabic and gelatin. Such zones are apparently similar to the above-described exclusion zones.

Also relevant is a somewhat later observation by Green and Otori [4], who produced results almost identical to our own. Exploring the basis of the so-called unstirred layer — the slow-diffusion region immediately surrounding many biological tissues [13] — these investigators exposed natural and artificial tissues to microsphere suspensions. The near-surface regions profoundly excluded the microspheres. Exclusion zones were typically several hundred micrometers wide, and could not be eliminated by vigorous stirring. The authors concluded that water in the near-tissue zone was physically different from water in the bulk, and that this difference could account for the long known and extensively studied “unstirred” layer that surrounds many if not all biological tissues (for review, [7]). Unfortunately, the authors apparently never went on to explore the basis for the observed near-surface difference.

The point is that although seemingly extraordinary, the claims above are not really new. Many investigators have produced ample evidence that interfacial water is profoundly different from bulk water. This evidence was not lost on many leading scientists. Nobel Laureate Albert Szent-Gyorgyi, the father of modern biochemistry, concluded that: “life is water dancing to the tune of solids.” Gilbert Ling, whose five pioneering books and numerous works spanned five decades, has emphasized interfacial water’s central role in all of cell function (e.g., [14]). Our own contributions stand on the shoulders of such giants [15]. As for the most recent experimental evidence from this laboratory, the reader is referred to the papers listed herein, as well as to a recent public award lecture [16]. This hourlong lecture concisely summarizes the basic as well as very recent evidence, and limns these findings’ unusually broad implications for science and technology. From this presentation, one can readily judge the strength of the evidence.

### 2.4. Exclusion Zone and Protons

A prime attribute of the interfacial exclusion zone is separation of charge. The zone itself is ordinarily negative, while the region beyond is positive. This positive potential is measured directly and is also consistent with pH measurements, which show an extreme drop of pH immediately beyond the exclusion zone, often to less than pH 3. This drop is observed not only with pH-sensitive dyes, but also with miniature pH probes [10]. Hence, so long as the exclusion zone builds, proton concentration will grow in the bulk water beyond.

But there is a physical difference between negative and positive zones. The negative charges are found within the exclusion zone, which is fixed in space. The positive charges, by contrast, are unconstrained and free to diffuse in the region beyond: These protons (or hydronium ions) will move depending on local electrical gradients. Essentially, these free positive charges arise as a byproduct of the environmental energy that builds the exclusion zone, and are free to accomplish whatever tasks their presence may require.

What might those tasks include?

### 2.5. Like-Likes-Like

In water, similarly charged particles *attract* one another. Feynman referred to this counterintuitive yet long-recognized phenomenon as “like-likes-like,” theorizing that the paradoxical attraction of like charged particles lay in abundant “unlikes” situated between the likes [17]. Thus, for negatively charged particles, the attraction would occur because of interposed positive charges, while for positively charged particles the required interposed charges would be negative.

Sogami and Ise [18], took a similar theoretical route, mainly based on the extensive experimental work by Ise and colleagues [19,20]. Ise’s experiments showed not only that like-charged colloidal particles attract one another, but also that as the particles drew closer, the attraction was increasingly balanced by an inter-particle repulsion; this balance yielded stable colloid crystals, within which elements were regularly spaced [21,22]. Again, the attractive force between like-charged entities was considered to arise as a result of unlike charges in between. And, an equally crucial feature of this attraction was the development of order.

On the other hand, the source of the required unlike charges has remained uncertain. Counter-ions have been considered as the likely source, but whether they might occur in adequate concentration has been less clear. A possibility that we investigated is that they might come from exclusion-zone formation. In the case of negatively charged entities such as the majority of biological substances, coalescence would require interposed positive charges. Thus, protons arising out of exclusion-zone formation could thus constitute the required “unlikes.”

How such a mechanism might work is as follows. Consider a suspension of negatively charged entities such as microspheres in water. As a result of incident radiant energy, each microsphere develops a negatively charged exclusion zone, thereby accruing free protons in bulk water. The exclusion zones take the form of microsphere-enveloping shells, while the protons are free to diffuse from these shells. The positive charges will then accumulate in between the microspheres, constituting the required unlikes that draw the microspheres toward one another. Coalescence continues until the attraction becomes balanced by inter-particle repulsion, at which point an ordered array is formed.

If light is the ultimate energizing agent, then by increasing the light intensity, more protons should be generated, and elements of the ordered crystal should draw nearer to one another. That is what the experimental results show: incident light diminishes the microsphere-to-microsphere distance [23].

A direct test of the like-likes-like-through-an-intermediate-of-unlikes hypothesis was carried out using ion-exchange beads [24]. Conducting this test was possible because attraction between beads could be seen even on large, experimentally accessible, scales. Thus, suspended in water, gel beads on the order of 0.5 mm diameter attracted one another at surface-to-surface separations as much as 400 μm. In the case of negatively charged beads, pH measurements showed a high proton concentration in between the beads, supported by microelectrode measurements that showed a relatively positive potential in the inter-bead region. Substituting positively charged beads for the negative ones gave a result that was polarity-opposite.

Hence, fresh experimental evidence confirms Feynman’s theoretical expectation: like-likes-like through an intermediate of unlikes. Like-charged entities attract; and, they become ordered.

### 2.6. Biological Coalescence

We return now to the issue at hand — the origin of life. The first step is likely to have been some kind of coalescence of substances. Substances initially widely dispersed must somehow have been brought together to form a proto-cell. How then might such coalescence happen?

A plausible explanation comes from the like-likes-like mechanism. Envision simple, identical molecules scattered widely about, dissolved in shallow water or in deeper water near a hydrothermal vent. These molecules could be simple sugars or short-chain amino acids — the latter being extremely primitive and found even in space and on the moon [25,26]. Radiant energy from the sun (or from a geothermal vent) beats down upon these primitive molecules. This energy builds exclusion zones around each one. If the exclusion zones are negatively charged, then their growth will generate free protons. These protons will then draw these molecules together, forming larger, ordered entities.

This kind of coalescence is experimentally confirmed. Radiant energy (heat) demonstrably causes amino acids to coalesce into highly ordered polyamino acids, called “thermal proteins.” These proteinlike structures in turn self assemble into distinct, microsphere-like entities [27], which easily classify as coascervates or gels. Such globular entities exhibit a striking array of cell-like features such as growth, breaking apart, selective accumulation and exclusion of organic substances, and even electrical action potentials [27-32]. Hence, they qualify as proto-cells.

Indeed, the polyamino-acid filaments of these protocells could well serve as primitive substrates for inheritance — as could the ordered water immediately surrounding them. Information is available, and could be passed along.

At the same time, proto-cells with differing polyamino-acid content could aggregate to form multiunit entities. Such entities would be similar to multi-cellular entities. Thus, a simple energy-based mechanism (solar or geothermal), operating through the like-likes-like principle, can produce coalescence, order, and even function at multiple levels of organization.

If these entities are produced by the like-likes-like principle, then they ought to contain large volumes of negatively charged, ordered water. This is direct expectation of the mechanism. The books by Pollack and Ling [14,15], review the evidence that both gels and cells are filled with ordered water. In our estimate the evidence is voluminous. Further, Oparin emphasized that simple coascervates are “swathed with a more or less thick membrane of water” and that this thick membrane was associated with negative charge [1]. This observation seems remarkably similar to that of the exclusion zone and the unstirred layer, and therefore lends strength to the presence of the like-likes-like mechanism.

Also of interest is the observation that the potential difference between the inside and outside of a gel/coascervate is similar to the potential difference between inside and outside of a cell. Their magnitudes are similar. From an electrical point of view, therefore, the membrane thus seems less consequential than thought. Given the negative electrical potential typical of most exclusion zones, a possibility is that the cell’s negative potential arises not from the membrane or its pumps and channels, nor even from its predominantly negatively charged proteins, but largely from the cell’s negatively charged water. Exclusion-zone potential can be up to 200 mV. This negative charge could account for much of the cell’s (and gel’s) negative electrical potential — which, interestingly, can be in evidence even after the cell has been de-membranated [33]. Hence, ordered water might play roles beyond that of bringing molecules into coalescence.

The emphasis so far has been on entities of like charge, which attract one another. This narrow focus should not be interpreted as suggesting that the ordinary attraction between unlike charges is not significant; in fact, it is central to the like-likes-like mechanism. The point is that whether entities are of opposite polarity or the same polarity, the evidence indicates that they will attract. Thus, counterintuitively, sun’s energy tends to drive all entities into coalescence.

For driving the coalescence necessary for life’s origin, then, a simple and primitive mechanism is available. Solar or geothermal radiation separates charge in water, which then draws dissolved or suspended entities into ordered coalescence.

### 2.7. Is Life’s Origin a One-time Event?

Although the exact date has been subject to occasional revision, the prevailing view is that life began some 3.5 billion years ago. A unique coalescence of events is thought to have taken place at that time, which gave rise to the first pre-cellular entity. Out of that entity grew life, as we know it.

Another possibility is that life continues to originate. If the above-mentioned condensation mechanism is indeed the first step, then that step might be occurring continually — for the required conditions are omnipresent. All that is necessary are primitive molecules, water, and the sun’s energy.

In such a scenario, the biosphere consists of living entities at various stages of evolutionary development. The most sophisticated we know well. The simplest are proto-cells that are no more complex than primitive gels or coascervates. Such proto-cells would then evolve with time into progressively more complex entities.

Quite remarkable is the extent to which such entities can resemble cells. Gels/coascervates can have negative electrical potentials just like cells; they absorb energy from the environment just like cells; and, like cells, their charged constituents move, here driven by energy from the environment. These constituents, including both water and dissolved/suspended substances, can be ordered, much like the cell. And, they can also grow like the cell: the like-likes-like mechanism expands their size so long as environmental energy is present to drive the expansion. Hence, these primitive gels/coascervates are more cell-like than one might initially suppose. The line of distinction between gels/coascervates and cells is somewhat blurred.

Thus, an issue to consider is how these primitive gels/coascervates might have begun evolving. Given the likely abundance of organic compounds on the early earth, chemically distinct populations of coascervates/gels might likely have been present early on. Through the like-likes-like mechanism, these populations could then have mixed, and coalesced. Over time, they might have undergone a process of chemical selection, in which those gels that could acquire beneficial characteristics might grow, break apart, re-grow, etc. Thus, the hypothetical pool could very well have worked like a combinatorial chemical process, in which distinct combinations were “tried” and only a few selected to dominate the early waters of earth. This is evidently speculative, but nevertheless a reasonable expectation of the coalescence mechanism here under consideration.

### 2.8. Membranes and Life

In considering the possibility that life’s origin might be an ongoing process, a question that inevitably arises is of the role of the membrane. The presence of an enveloping membrane is considered so essential that often it is invoked as a defining feature of life. After all, what could be more fundamental than the cell membrane?

A problem is how the membrane might have come into being in the first place. The appearance of a simple phospholipid membrane enveloping the coascervate/gel could have occurred as a chance event. But, if so, it would have posed an immediate and urgent problem, for the enveloping membrane cuts off the easy interchange between the cell and its surrounding environment. The cell would be in serious trouble. The solution, of course, is to add trans-membrane conduits such as ion-specific channels, pumps, and other membrane-based entities that can perform the task of inside-outside transport. The existence of such entities is widely acknowledged.

However, there is a problem of timing. Unless the above-mentioned entities were created contemporaneously with the new membrane, the membrane-enveloped cell would choke. These conduits would have to have appeared when the membrane appeared. But what might have been the pressure to create such entities? Or, indeed to create the genetic apparatus required for their manufacture? To our knowledge, this question has never been satisfactorily answered. It poses a dilemma.

Extricating ourselves from the horns of this dilemma could reside in downgrading the significance of the membrane. For example, if the membrane were something less than a *continuous* barrier, then the need for special membrane-based assets would be less acute; inside-outside exchange would not be compromised. Recall that the need for an all-enveloping barrier initially stemmed from the perception that in the absence of such a barrier, cellular contents would disperse. With the mechanism under consideration here, however, there is no such problem: the gel/coascervate sticks together naturally, by energy-induced coalescence. A continuous membrane is unnecessary. This argument should not be taken to imply that no membrane is present around the contemporary cell, but only that absolute continuity is neither necessary nor even advantageous.

The possibility that the membrane might be discontinuous would appear to fly in the face of convention, for membrane continuity is considered essential for cell function. The book by Pollack [15], however, gives six different examples of seemingly normal cell function in the presence of disrupted membranes. Cells perform their routine tasks even when their membranes contain substantial holes and in the demonstrable absence of re-sealing. For those presuming that membrane integrity is essential to life, this conflicting evidence needs to be examined, and dealt with.

On the other hand, if cell membranes are ordinarily discontinuous or leaky, then the limited impact of any further disruption might be anticipated; even when challenged by larger membrane breaches, the cell might still function. As a gel, the cytoplasm would remain intact. The electrical potential would still persist, and whatever order existed in the cell could well remain. In other words, the breach could be of only secondary significance. By this presumption, the above-mentioned dilemma would resolve, for never would any acute need have arisen for the cell to quickly manufacture functional entities for which the required machinery could not reasonably have been available. The proto-cell would in fact more closely resemble the cell.

## 3. Conclusions

Drawing a “conclusion” about something as seemingly remote as life’s origin is not simple; one can merely speculate on the factors that might have been responsible — and might still be responsible — for the creation of life.

The situation is made difficult by the lack of an accepted definition of life. Some argue that life is defined by the presence of a membrane-enclosed cytoplasm; others argue that the basis must include division and reproduction; and still others argue in terms of energetics, that life is an ordered system and hence full of configurational potential energy. Others argue that there is no life without motion, and hence generated motion is most basic for defining life. The list goes on.

While some of those features are implicitly included in the mechanistic construct derived here, a central feature of this construct is the presence of order, particularly ordered water next to interfaces. Ordered, exclusion-zone water is fundamental to all arguments put forth; without this negatively charged water, there would be few “unlikes” and hence limited attractive forces needed to bring likecharged entities into coalescence. There would be no life.

Indeed, the generated protons may have more than one function. Besides creating the attractive forces responsible for coalescence, they may also create movements of other kinds such as steady flows and osmotically driven flows [34,35]. Thus, the free protons created ultimately from environment-supplied energy may be functionally versatile.

Likewise, a critical role may also be played by the negative charge. Although this charge is embedded in the ordered water adjacent to the hydrophilic interface, constituent electrons are not necessarily fixed; they can move about within the confines of exclusion zone, much like charges in a semiconductor. The fact that the exclusion zones fluoresce [36], implies that constituent electrons can move from higher to lower energy states. Hence, exclusion-zone electrons may be free to partake of any process requiring energetic electrons.

One such process is metabolism. Oxidation of foods involves electrons moving down a chain from higher to lower energetic states, and an ample source of such electrons is the exclusion zone. Indeed, the ability of gels to transfer electrons to organic compounds amounts to oxidation. A rudimentary “metabolism” could thus arise in these simple, organic compound-containing gels.

In other words, the charges are versatile. Exclusion-zone electrons may be involved with metabolism, while exclusion-zone-generated protons may be involved with motion. Separated by energy from the environment, these charged entities would take on distinct but critical roles even in the primitive cell or proto-cell. After all, without metabolism and motion, there can be no life.

Thus, one additional definition of life can be thrown into the hopper of options: energy-driven charge separation. The environment supplies the energy, while interfacial water is the substrate for charge separation. This definition implicitly includes several of the other ones mentioned above. Also, it puts water at the center of life, which serves in turn to make clear why life is not possible in the absence of water. Life almost certainly originated (or, is originating) in water, and life cannot go on without water.
